# Patients' Perspectives on Postoperative Follow‐Up Calls in Day Surgery: A Qualitative Study

**DOI:** 10.1111/scs.70077

**Published:** 2025-07-01

**Authors:** Mira Rajala, Maria Kääriäinen, Anitta Tanhua, Pirjo Kaakinen

**Affiliations:** ^1^ Research Unit of Health Science and Technology, Medical Research Centre Oulu, Faculty of Medicine University of Oulu Oulu Finland; ^2^ The Finnish Centre for Evidence‐Based Health Care: A Joanna Briggs Institute Centre of Excellence Group Helsinki Finland; ^3^ Oulu University Hospital Oulu Finland; ^4^ Wellbeing Services Country of North Ostrobothnia Oulu Finland

**Keywords:** day surgery, development of care, follow‐up calls, postoperative guidance, qualitative research, telephone nursing

## Abstract

**Aim:**

To describe the day surgery patients' perspectives on postoperative follow‐up calls.

**Background:**

Follow‐up calls allow for providing information, advice or help managing the patient's well‐being after surgery at home. They are an easily accessible way to provide support regardless of geographical location. Day surgery units often do not have a routine for systematic postoperative follow‐up, and there is no research on postoperative follow‐up calls from the patient's perspective.

**Method:**

A descriptive qualitative study was used. The data were collected in August–September 2020 through face‐to‐face thematic interviews with one university hospital's day surgery patients and analysed using inductive content analysis. COREQ guidelines were followed.

**Results:**

Thirty‐one patients from various surgical specialties were interviewed. Four main categories were identified from the perspectives of postoperative follow‐up calls: being able to plan the call, expected benefits to patients, expected benefits to healthcare and expected content for the follow‐up calls. Patients perceived postoperative follow‐up calls as necessary; it is seen to develop the treatment process and the continuity of treatment, supporting self‐care and providing safety for self‐care at home after surgery. Patients also suggested content areas to be considered during follow‐up calls.

**Conclusion:**

Patients feel that maintaining contact with the surgery unit after discharge is essential. The results show that follow‐up calls could be an efficient guidance and support method after day surgery. From the patient's perspective, they may allow for individual attention, potentially prevent unpleasant feelings and risks related to the disease and its treatment, support self‐care and increase the continuity of care.

**Relevance to Clinical Practice:**

The results show that patients, regardless of age and background, are ready to receive guidance and support by telephone and know how to utilise the treatment method efficiently. The results support integrating follow‐up calls as a natural part of healthcare patient guidance and support methods.

## 1 Introduction

Day surgery, when the operation is performed without having to stay in the hospital for more than 24 h [1], is a safe and cost‐effective treatment [2]. Patients are delighted with day surgery, and their experiences are overwhelmingly positive [3, 4]. Patient satisfaction in surgical treatment is significantly related to the degree of preparation of patients for surgery and discharge [4, 5], and no significant difference in patient satisfaction has been observed between the in‐patient and out‐patient groups [6].

Telephone nursing is a practice where healthcare professionals provide patients with guidance and support regarding treatment and care over the telephone [7]. This practice benefits healthcare by improving care efficiency, speed and availability. Telephone nursing helps patients receive the right advice and care at the right time, ensures continuity of care and refers patients to local care if necessary [8]. It can also potentially reach many underserved patients cost‐effectively [9]. Although face‐to‐face guidance is superior—particularly for dietary compliance and physical activity—telephone‐based intervention has yielded comparable or even better results in diabetes guidance, glucose monitoring, foot care and cholesterol‐level guidance [10]. Personal support during telephone nursing increases self‐efficacy and reduces the risk of adverse clinical events in patients suffering from hypertension [11], chronic heart failure [12] and/or COPD, demonstrating that collaboration and guidance between patients and healthcare professionals can be achieved without a face‐to‐face consultation, even among vulnerable patient groups [13, 14].

Patients are vulnerable soon after discharge, so post‐discharge guidance and support can improve clinical outcomes [15] and enhance patient satisfaction with the clinical care experience [16]. The study shows that guidance and support related to disease and treatment positively affect the patient's quality of life after surgery [17]. Guidance and support aim to promote changes in health behaviour [18] and help patients understand the importance of behavioural risks. Patient‐centred guidance and support recognise that the motivation for change must come from the patient and their needs [19]. Common obstacles to patient‐centred guidance and support include preconceived notions about the patient's role in their care and a relationship in which the guidance and support provider is viewed as an expert while the patient is expected to follow instructions [20]. Involving the patient and evaluating their perspective has been essential in offering patient‐centred functions and implementing care to promote patient health [21].

Outpatient care facilities must monitor and assess their practices, promptly respond to patient needs and consider the perspectives and opinions of patients when developing daily operations [22]. Both health and guidance literature on telephone‐based care is scarce [23], underscoring the need for further research into telephone nursing, including its effectiveness [24]. Additionally, there is a lack of information on postoperative follow‐up calls in day surgery from the patient's perspective. Day surgery units often lack a routine for systematic postoperative follow‐up, and patients are usually expected to manage their recovery after surgery [25]. Patients have also reported difficulties contacting healthcare for professional support following surgery [26]. Therefore, the purpose of this study was to describe day surgery patients' perspectives on postoperative follow‐up calls. The aim was to generate new information for developing patient‐centered postoperative telephone nursing and enhancing the continuity of care and patient recovery after surgery.

The research question was: What perspectives do day surgery patients have on postoperative follow‐up calls?

## 2 Methods

### 2.1 Design

A descriptive qualitative study was used to identify information on patients' perspectives [27]. The Guidelines of The Consolidated Criteria for Reporting Qualitative Research (COREQ) [28] were followed in reporting.

### 2.2 Participants

Day surgery patients were selected using purposive sampling to obtain information relevant to the study [27]. Recruitment was conducted through the day surgery unit of the University Hospital's Outpatient Clinic. The inclusion criteria for participants were as follows: they had to be ≥ 18 years old and receive preoperative telephone nursing according to the organisation's procedures 1 week before their scheduled surgery. Although the interviews included questions about preoperative telephone nursing, the current study did not utilise the related findings. However, patients' familiarity with the preoperative telephone nursing process might have helped them articulate their perspectives on implementing postoperative follow‐up calls.

### 2.3 Data Collection

The data were gathered in August–September 2020 through face‐to‐face thematic interviews. The interview framework was pre‐tested with the first interviewees, and since there were no changes, their interviews were also included in the study. The researcher (MR) interviewed the patients on the morning of their surgery in the quiet office of the day surgery unit when the patients were easily accessible, eliminating the need to schedule a separate interview time. Apart from the interviewee and the researcher, no other participants, such as loved ones or healthcare staff, were in the room. Initially, the patients were briefly informed at a general level that during the postoperative follow‐up calls, they would be reached at home after surgery to assess their overall condition and recovery. The interview questions were quite general, thereby not steering the patients' views, such as: *What kind of views do you have about postoperative follow‐up calls? What should be considered when implementing postoperative follow‐up calls? What benefits or challenges do you think are related to postoperative follow‐up calls? What content would be beneficial to cover in follow‐up calls?* The interviews were audio recorded with the consent of the interviewees. The researcher also took field notes during the interviews, which partially aided in recalling the interview situations, though their content was not necessary for the analysis. The interviews continued until saturation had been reached after a few interviews, leading to a final target group of 31 patients. There was also no need for follow‐up interviews. The total data collection time was 7 h and 45 min, with an average interview duration of about 15 min. The transcribed texts totalled 206 pages (Times New Roman, 12 font, 1.5 line spacing). The transcriptions were not returned to the patients for comments, as it was promised that their exact contact information would be destroyed after the interviews.

### 2.4 Data Analysis

Inductive content analysis was utilised [26], allowing the research to proceed entirely based on the interview data. No software was employed for data management; instead, the transcribed data were structured and analysed manually using a standard word processing program. The analysis began with multiple readings of the data. The unit of analysis included words, sentences and ideas that addressed the research question. Initially, the original quotes from the interviewees were simplified (*n* = 208), and those with similar content were colour‐coded. Ultimately, the condensed statements with the same content were organised into subcategories (*n* = 17) and combined into four main categories. The researchers revisited the data throughout the analysis to confirm that the formed categories aligned with their content. The interviewer conducted the initial analysis, followed by a review from two additional researchers who made necessary revisions and adjustments to enhance the findings. Once the analysis was concluded, an expert from the target organisation further scrutinised it to ensure that the overall context and abstraction accurately represented the day surgery setting. An example of the analysis is presented in Table A1.

### 2.5 Ethical Considerations

The ethical principles of the research were maintained throughout all phases of the study [29]. Appropriate permits were obtained from the target organisation, and a favourable opinion was sought from the Ethics Committee of the Hospital District (42/2017). The researcher informed patients about the study, ensured their informed consent to participate, and clarified the rights of participants during the research. None of the interviewed patients declined to participate or withdrew from the study. The research complied with the European Union's General Data Protection Regulation [30].

## 3 Results

Half of the interviewees were women (52%). The age range was 22–82 (average age 55.3). Table A2 presents detailed demographic characteristics of the interviewees (*n* = 31).

The day surgery patients' perspectives on postoperative follow‐up calls were formed into four main categories: *being able to plan the call*, *expected patient benefits*, *expected healthcare benefits* and *expected content for the follow‐up call*.

*Being able to plan the call* included subcategories *anticipation of the call*, and *timing of the call* (Table A1). In *anticipating the call*, it was considered essential to announce and agree in advance when the postoperative follow‐up call will take place.

Well, yes, then, of course, could announce approximately the time when they will call. It would be important to know in advance, yes. (M79)

The *timing of the call*, about a week after surgery, came up the most. Patients felt that pain and other symptoms were still intense in the initial stages of recovery, so after a week, the situation would stabilise more, and the recovery process would be more coherent. During the week, patients thought they had time to think about things they could clarify in a follow‐up call. It was felt that possible written inquiries come a long time after the surgery when they are no longer relevant.

I think that probably a week could be a good time because earlier it's probably just like the pain is so bad. After a week, I probably would have had time to try what I could do. And then I guess I have this surgery, so even in a week, I will hardly be interested in going anywhere to ask questions or visit anywhere if I don't absolutely have to. (F48)

*Expected patient benefits* included subcategories *importance*, *sense of care*, *sense of security*, *reduction of uncertainty*, *increasing confidence*, *continuity of care*, *possibility of discussing with a specialist* and *effective problem‐solving*. The *importance* of postoperative follow‐up calls included patients' aspects such as meaningfulness, necessity, effortlessness and unnecessary calling. Patients found postoperative follow‐up calls meaningful and essential, suggesting immediate implementation. They had the experience of a follow‐up call from a private hospital, and these calls were positively received. Since patients often hesitate to seek help with unclear issues, calling after surgery was seen as beneficial. The necessity of follow‐up calls was highlighted to support patients' post‐surgery, and patients felt that resources should be allocated for this. Effortlessness manifested to patients so that the call home was not perceived as disturbing or harmful. The calls were seen as a valuable service. However, the patients deemed the calls unnecessary if no issues arose post‐surgery. They did not identify any challenges or negative aspects of postoperative follow‐up calls, even though they were asked about them separately.

Very good. It is essential to call, especially those who don't know, so if there's a concern about this or that, and how you've been coping and so on, it's excellent if you ask how you're doing. Well, at least I don't dare bother anyone with my questions. It's a good thing that calls to ask how you're doing… (F43)

Patients felt that postoperative follow‐up calls would enhance their *sense of care*, as healthcare staff would demonstrate interest in their well‐being. This would create a sense of comfort when a nurse calls to check on their condition. Patients expressed that there is currently a perception in healthcare that they are not always adequately cared for, so this approach would strengthen the feeling that patients are not alone in their illness or recovery. They believed that follow‐up calls would improve patients' *sense of security*, as there might not be anyone knowledgeable at home. They would attempt to manage independently in uncertain situations without the confidence to call. Patients shared that this could reduce unnecessary worry, as they would know that a nurse would contact them after surgery.

Not just that the patient comes, goes, bye, but a follow‐up call would let me feel that I am taken care of…this unit is further away; it's nice that they take care of how you're doing after surgery. (F43)

Patients believed follow‐up calls after surgery would *reduce the uncertainty* surrounding their surgery, recovery and at‐home management. They feel that a conversation with a specialist over the phone could help ease this uncertainty.

I have many friends who have undergone this same procedure. And everybody is always about, eh, are these sensations normal? Is it ok now if I feel a little pain but not in a normal way? Is this serious, and is this skin now going to gangrene or not? And terrible uncertainty is often associated with this procedure. (F26)

Follow‐up calls were also thought to *increase confidence*, especially in managing post‐surgery life at home. Patients believed it would enhance the *continuity of care*, ensuring that treatment extends beyond the surgery and continues at home. This would sustain the connection with the surgery unit. It was considered that continuity of care is particularly important for the elderly, who may face more challenges after surgery and are not always proactive in seeking help, often just hoping for the best in their recovery. Patients stressed the necessity of not being forgotten after discharge and valued ongoing support during their recovery. They felt that although they were given a telephone number to call if needed or advised to contact the health center, the connection with the surgery unit ceased with discharge. In prior surgery experiences, patients felt that they had been left alone and would have appreciated someone checking on them after discharge.

When I, too, have been operated on several times, I always felt like I was left alone. (M69)

… especially older people can be like that: they suffer alone without any connections…it's a bit like what has been done and how it will continue… it adds to the continuity of treatment. There is such practice as being at home, managing alone, and contacting the local health center if you need something. (F58)

Considering the *possibility of discussing with a specialist* was deemed an essential part of postoperative follow‐up calls, particularly for those living alone. Patients value the opportunity to ask questions, respond to specialists' inquiries and tackle broader issues beyond immediate surgery. They believe that, as laypersons, they might not grasp all the crucial post‐surgery details, allowing a specialist to provide a broader perspective. This channel enables patients to inquire about topics they might otherwise hesitate to discuss. Postoperative follow‐up calls are perceived to allow patients to think about their questions in advance. Often, inquiries arise later at home, and having a specialist familiar with their case proves beneficial. Patients indicated that postoperative challenges can occur, making discussions with specialists vital. They prefer the specialist to be well informed about their situation before the call, thus avoiding repetitive explanations to different healthcare personnel who lack prior knowledge of the case.

…And you know that if you have something to ask, you can prepare for it, and if you are unsure about something, you can ask about it. (F79)

It could be the nurse, who would have been familiar with the matter before so that you don't have to explain from the beginning in another place. The caller also knows the patient, so it feels good. (F58)

Patients felt that *problem‐solving* would be *effective* in follow‐up calls, enabling them to address issues and receive new instructions, reassurance and support. From the patients' perspective, follow‐up calls can efficiently tackle these issues by quickly checking their status and keeping the calls brief if they feel fine. This method facilitates easy and effective communication and quickly raises potential concerns. According to patients, it provides an opportunity to discuss minor issues without needing to call or visit the health centre. Without it, they tend to manage especially minor concerns independently without seeking help.

Well, if there happen to be any problems. You can bring them up and just go through things quickly and get help. (M73)

*Expected healthcare benefits* included subcategories *development of the treatment process*, and *reduction of the burden on healthcare organisations*. Patients felt that postoperative follow‐up calls were seen to *develop the treatment process*, improving care quality and enhancing patient outcomes.

I would perceive it as this kind of care and that they really care about the patient. In healthcare, I feel that nowadays, there are so many bad things that there are those who don't care. …that's what people in healthcare should do and develop the treatment process… They have to take care of the customer and the patient. (F26)

According to patients, follow‐up calls can help *reduce the burden on healthcare organisations* by minimising post‐surgery visits for questions or unclear issues. It was felt that patients often don't know who to contact, leading to strain on various healthcare services.

Just leave you at home to manage there, contact the local health center if you need something, and then all the patients will call everywhere and even the emergency room, where they could get help. (F58)

*Expected content for the follow‐up call* included subcategories *wound care*, *pain management*, *activity methods*, *coping at home* and *general condition*. Day surgery patients felt that postoperative follow‐up calls should consist of *wound care* checks, as they may lack knowledge about routine wound healing. Patients felt that they might worry about proper healing and care. They believed that follow‐up calls could reassure them about the healing process and inform them about the symptoms of infection and signs of wound necrosis.

It would certainly be excellent because there will certainly be some who wonder if this is normal wound healing. At the very least, you will be assured of the healing and treatment of the wound. You don't have to think and reflect alone. (F28)

It was felt that assessing pain intensity, continuity and home treatment methods for *pain management* was vital. Some patients have experienced severe pain post‐surgery, where they would have needed specialist advice. Patients felt that pain experiences vary and are individual, so checking the adequacy of painkillers is essential.

…because all people are so individual, and for everyone, Panadol or Ibuprofen isn't enough for the pain. And then if they are at home, not everyone can tell the doctor about the pain and visit the doctor… (M41)

It was felt that post‐surgery may be uncertain about correct *activity methods* and should discuss their practices with a specialist. From previous experiences, some patients recall attempting exercises despite severe pain, unsure if it was acceptable.

…like that, then there would probably be questions about movement and activities: what can I do and how should I move? (F48)

The patients considered it essential to ask about overall *coping at home*, check for any problems arising, and map out potential help needs. Checking the patient's *general condition* and well‐being post‐surgery was also mentioned as necessary. Ensuring that someone inquires about their health and recovery progress at home was felt to provide valuable information and help ensure the success of the surgical outcome.

So that someone asks how you're doing, how you're feeling in general, how is your condition, and how it's been. Yes, it might involve everything if you're in severe pain, and it affects your health in general. That way, you can talk about it then. It would be a good thing. (F63)

## 4 Discussion

Day surgery units often lack a routine for systematic postoperative follow‐ups [25], and there is limited comprehensive information on this topic [22]. Therefore, the study aimed to generate new insights to enhance postoperative telephone nursing, improve continuity of care, and assist in patient recovery after surgery. It focused on incorporating patient perspectives to ensure patient‐centredness when developing postoperative follow‐up calls within the context of day surgery. The study also sought to understand how day surgery patients perceive the importance of these follow‐up calls and to identify the content they consider essential to include.

Both face‐to‐face and telephone nursing can significantly influence treatment outcomes. However, the results that have been found to achieve better outcomes have varied regarding different diseases and the goals of guidance [10, 11, 12, 13, 14]. Compared to face‐to‐face and telephone nursing in the postoperative follow‐up of day surgery, patients preferred the telephone nursing option, perceiving it as having a higher overall satisfaction. They felt it offered effective postoperative mapping, allowed patients to voice their concerns, and was generally more convenient [31]. Patients in this study emphasised the importance of immediately implementing postoperative telephone nursing, seeing it as a step to enhance the overall care process and improve the quality and functions of care. Furthermore, according to previous research, implementing postoperative follow‐up calls can alleviate additional work for hospitals, improve patient outcomes [32], help reduce healthcare costs [31, 32] and save time in guidance [31]. Most patients could benefit from minimising early readmission if the follow‐up calls process is optimised to ensure routine and timely follow‐up calls are made to all discharged patients [32]. Additionally, patients in this study believed that postoperative follow‐up calls could decrease post‐surgery visits and calls to health centres.

In postoperative follow‐up calls, announcing the implementation time in advance was deemed essential, with about a week after surgery being most frequently mentioned. At this point, the situation (e.g., pain and other symptoms) has typically stabilised, allowing for a more precise assessment of recovery progress. Follow‐up calls by a nurse early in the surgical recovery process may be necessary to educate patients about recovery; however, contacting patients right around the time of surgery may be too early for detailed guidance [33, 34, 35]. A previous study found that patients were reluctant to receive additional instructions on the first day because they were ‘tired’. Patients could start reflecting on their experience about 3 days post‐surgery and reported feeling a sense of healing. Although they were still in pain and some felt distressed by managing their recovery independently, they appreciated the opportunity to express their concerns to a nurse about the overall experience. At this stage, they also began asking questions regarding potential issues, and most care‐related inquiries were addressed [35]. When patients were allowed to contact the unit by telephone, 62% reached out during the first postoperative week [34].

Patients and their loved ones may experience a lack of guidance, support and insecurity after discharge due to decreased contact with the surgical unit, leaving patients to manage the recovery process independently [25, 26, 36]. A previous study has shown that postoperative guidance and support positively affect the patient's quality of life after surgery [17], and this study found similar insights. According to the patients in this study, postoperative follow‐up calls can enhance the perception that healthcare staff are interested in the patient and their well‐being. Feelings of safety and self‐confidence can also increase, reducing uncertainty. Postoperative follow‐up calls were perceived to improve the continuity of care, ensuring that treatment continues even after surgery and maintaining a connection to the surgical unit. This was regarded as especially important for older patients, who may face more significant challenges in recovery. It was also noted that guidance and support after surgery are crucial for those living alone to establish a conversational connection. Patients felt they could discuss their experiences during a postoperative follow‐up call and address important or unclear issues. According to patients, the caller should familiarise themselves with the patient's situation in advance to provide individual, patient‐centered guidance and support. This approach also prevents the patient from repeating the same information to multiple people.

Surgical patients' self‐care is linked to the management and prevention of postoperative symptoms, which include healthy nutrition, skin care, mobility, and the ability to respond to common symptoms, such as post‐surgery fatigue and pain, as well as wound care management and knowledge of how and when to seek healthcare support [37]. A previous study indicated that the most frequent reasons for patient contact were surgical wounds and pain [34], a theme that also emerged in this study. Patients believe that wound healing and care should be evaluated during postoperative follow‐up calls since they may lack experience with the normal wound healing process and its treatment. This practice also allows for early detection of inflamed or improperly treated wounds. Regarding pain management, it is crucial to assess the intensity and continuity of pain along with its treatment methods, acknowledging that pain experiences are always individual. A previous study found that 6 months postoperatively, a quarter of all patients reported wishing they had received more information on pain management [38].

In this study, there was concern about potential uncertainty regarding the appropriate recovery behaviour after surgery, and the patient may not be aware of misbehaving; in this context, postoperative follow‐up calls can be beneficial. Any recovery‐related issues encountered at home can be addressed, and solutions may be obtained. Furthermore, the patient's condition, endurance and well‐being can be assessed, as well as their home environment. Although nutrition, fatigue and sleep were not mentioned in this study, they can easily connect to the concepts of well‐being. However, these factors are crucial to recovery and provide significant insights into the patient's overall well‐being, making it essential to address them during the call [38]. The interviews did not discuss considering loved ones, but their involvement could enhance patient‐centered care [39]; loved ones may also require support following discharge [36].

The interaction between professionals and patients regarding health and treatment, respecting patient preferences and providing emotional support, along with processes that impact continuity of care and discharge planning, has emerged as important intervention areas [39]. Developing telephone nursing in healthcare is essential, as it can enhance the quality and efficiency of care, reduce hospital visits and improve health outcomes [40]. Standardising and integrating follow‐up calls into existing healthcare frameworks could involve training healthcare professionals according to unified guidelines and utilising technology such as electronic health record systems [41]. Additionally, emphasising patient‐centred care in telephone nursing could ensure that each patient's unique needs and preferences are considered [19], leading to more personalised and effective healthcare [32]. Implementing telephone nursing may require initial investments in training and technology; however, it can ultimately save personnel resources by streamlining processes and reducing the need for post‐surgery visits and calls to health centres. This could also support healthcare policy goals, such as reducing costs [31, 32] and improving access to care [32]. For this reason, it is crucial to investigate the future effects of telephone nursing in various contexts, examine its advantages compared to the current patient guidance and support, and consider healthcare professionals' perspectives.

### 4.1 Strengths and Limitations

The reliability of the research was assessed by examining credibility, confirmability, transferability, dependability and authenticity [27, 42]. The study's credibility is enhanced because the interviewees had recent experience implementing preoperative telephone nursing, so they were familiar with the phenomenon of telephone nursing [27]. The interview questions were pre‐tested and discussed with other researchers to ensure that the researcher's perspectives did not influence the interviewees. The detailed descriptions of the interviewees and research stages improve the results' confirmability. Initially, the interviewer independently conducted the coding and analysis. Subsequently, two other researchers (PK, MK) reviewed and refined the analysis to emphasise the key aspects of the research topic. A representative and specialist from the target organisation verified the analysis and findings to mitigate bias. The practices in different countries and day surgery units vary, limiting the transferability of the research results; however, the findings are not strongly tied to the operating principles of a specific organisation. To enhance reliability and authenticity, authentic expressions of patients are prominently featured in the results, and efforts have been made to underscore their connection to the research findings.

## 5 Conclusion

By involving patients in the development work from the start, it is possible to create patient‐centered methods that are accessible and usable while also making patients aware of their importance in the treatment process. The results of this study indicate that patients feel the need for contact following surgery, and they perceive communication with the surgical unit during the initial discharge phase as essential. Participants in this study believe that follow‐up calls can serve as an effective and practical guidance and support method after outpatient surgery. From the patient's perspective, this approach fosters individualised attention, alleviates unpleasant feelings related to the disease and its treatment, enhances self‐care and promotes continuity of care. The content of postoperative follow‐up calls for outpatient surgery addresses the informational needs of patients recovering from surgery. Based on the feedback from patients and healthcare personnel, it is possible to create comprehensive, practical, cost‐effective and adaptable telephone nursing programs tailored to the unique needs of various patient groups. For telephone nursing to become a natural aspect of patient guidance and support, healthcare professionals should receive training on the principles and practices of telephone nursing. The perspectives of the professionals involved should also be researched and considered.

## 6 Relevance for Clinical Practice

Day surgery patients often experience uncertainty after discharge and feel that they may be left alone without contact from the treatment unit. For this reason, future efforts should focus on developing postoperative patient guidance and support interventions. The results indicate that patients, regardless of age and background, are willing to receive guidance and support via telephone and know how to use the treatment contact provided by phone effectively. Generally, postoperative follow‐up calls can help secure the recovery process for patients of all ages, support post‐surgery care at home and identify patients who need additional help or follow‐up care. This study offers a comprehensive overview of patients' perspectives on follow‐up calls in day surgery, which can enhance the confidence and competence of healthcare staff in implementing telephone nursing. The findings advocate integrating telephone nursing as a standard part of patient guidance and support methods in inpatient and outpatient settings [6]. The insights from this study regarding postoperative follow‐up calls can be applied in planning and executing telephone nursing and follow‐up calls.

## Author Contributions

**Mira Rajala:** conceptualization, resources, investigation, visualisation, methodology, project administration, writing – original draft, writing – review and editing. **Maria Kääriäinen:** conceptualization, supervision, visualisation, methodology, writing – review and editing. **Anitta Tanhua:** writing – review and editing. **Pirjo Kaakinen:** conceptualization, supervision, visualisation, methodology, writing – review and editing.

## Ethics Statement

As part of a larger study, this research received approval from the Ethics Committee of the Hospital District (42/2017). Participants gave their informed consent.

## Conflicts of Interest

The authors declare no conflicts of interest.

## Data Availability

Research data are not shared.

## Appendix A

**TABLE A1:** An example of analysis: Original quotes and subcategories from day surgery patients (*n* = 31) under the main category ‘Being able to plan the call’.

Original quotes	Subcategory	Main category
‘Well, yes, then, of course, could announce approximately the time when they will call. It would be important to know in advance, yes.’ (M79) ‘The postoperative call should be known in advance.’ (M54) ‘Once it has been agreed to call in advance, it is a good thing.’ (F28)	Anticipation of the call	*Being able to plan the call*
‘I think that probably a week could be a good time because earlier it's probably just like the pain is so bad. After a week, I probably would have had time to try what I could do. And then I guess I have this surgery, so even in a week, I will hardly be interested in going anywhere to ask questions or visit anywhere if I don't absolutely have to.’ (F48) ‘Postoperative follow‐up call a week after surgery would be good because then you will no longer be happy to go anywhere to ask about unclear matters unless you have to.’ (F28) ‘A week after surgery would be a good time for a postoperative follow‐up call because the situation has stabilised somewhat, and you can better report on the progress of your recovery at home.’ (M70) ‘A week after surgery would be a good time for a postoperative follow‐up call.’ (F53) ‘During the week, you have already had time to think about questions that can be asked in a postoperative follow‐up call.’ (M79) ‘A week after surgery would be a good time for a postoperative follow‐up call.’ (F82) ‘Possible written inquiries after surgery have come late so that the call would come at the right time.’ (M69)	Timing of the call	

Abbreviations: F = female; M = male; Number = age.

## Appendix B

**TABLE A2:** Demographic characteristics of the interviewed day surgery patients (*n* = 31).

Variable	Frequency	Percentage	Mean
Gender			
Male	15	48	
Female	16	52	
Age			
Less than 65y	19	61	
65y and older	12	39	
Average age			55.3
Surgical specialty			
Orthopaedics and traumatological surgery	15	48	
Hand surgery	4	13	
Vascular surgery	3	10	
Gastroenterological surgery	3	10	
Neurosurgery	2	7	
Plastic surgery	1	3	
General surgery	1	3	
Cannot say	2	6	
Number of day surgery			
First surgery	15	48	
Second surgery	4	13	
Third surgery or more	11	36	
Cannot say	1	3	
